# Assessing baloxavir susceptibility of influenza viruses circulating in the United States during the 2016/17 and 2017/18 seasons

**DOI:** 10.2807/1560-7917.ES.2019.24.3.1800666

**Published:** 2019-01-17

**Authors:** Larisa V Gubareva, Vasiliy P Mishin, Mira C Patel, Anton Chesnokov, Ha T Nguyen, Juan De La Cruz, Sarah Spencer, Angela P Campbell, Mallory Sinner, Heather Reid, Rebecca Garten, Jackie M Katz, Alicia M Fry, John Barnes, David E Wentworth

**Affiliations:** 1 Influenza Division, National Center for Immunization and Respiratory Diseases, Centers for Disease Control and Prevention (CDC), Atlanta, United States of America; 2 Battelle Memorial Institute, Atlanta, United States of America; 3 Illinois Department of Public Health, Springfield, United States of America

**Keywords:** Cap-dependent endonuclease inhibitor, polymerase acidic protein, influenza surveillance, influenza, drug resistance, phenotypic testing, next generation sequencing

## Abstract

The anti-influenza therapeutic baloxavir targets cap-dependent endonuclease activity of polymerase acidic (PA) protein. We monitored baloxavir susceptibility in the United States with next generation sequencing analysis supplemented by phenotypic one-cycle infection assay. Analysis of PA sequences of 6,891 influenza A and B viruses collected during 2016/17 and 2017/18 seasons showed amino acid substitutions: I38L (two A(H1N1)pdm09 viruses), E23G (two A(H1N1)pdm09 viruses) and I38M (one A(H3N2) virus); conferring 4–10-fold reduced susceptibility to baloxavir.

On 24 October 2018, the United States (US) Food and Drug Administration (FDA) approved a new anti-influenza therapeutic, baloxavir marboxil, following its approval in Japan earlier in the same year [1,2]. This antiviral is prescribed as a single tablet for treating acute uncomplicated influenza A and B infections in patients 12 years and older [3]. In clinical trials, treatment-emergent amino acid substitutions (AAS) in the polymerase acidic (PA) protein causing reduced susceptibility to baloxavir have been reported at the rate of 2-20%, depending on age and other factors [4-7].

Early detection of emerging resistance is essential for timely modification of public health policies and recommendations on the use of antiviral therapeutics. Therefore, our aim was to determine baloxavir susceptibility of seasonal viruses before the drug entered the US market and establish a methodology to conduct baloxavir surveillance.

## Amino acid substitutions associated with reduced susceptibility to baloxavir

Baloxavir marboxil is metabolised to baloxavir acid, a potent inhibitor of the cap-dependent endonuclease of the PA protein. It halts viral mRNA synthesis, thereby stopping the early stages of virus replication [8]. Treatment-emergent reduced susceptibility was associated with AAS at residue 38 in the PA [4-7]. Viruses engineered to contain one of the three principal AAS (I38T, I38M or I38F) displayed 2–57-fold reduction in susceptibility, with I38T conferring the highest fold change [5]. The effects of these substitutions on drug susceptibility were type- and subtype-specific [5]. In addition, other AAS within the PA endonuclease active site were detected and investigated. Currently, there is no guidance issued by the Expert Working Group on Antiviral Susceptibility for the World Health Organisation (WHO) Global Influenza Surveillance and Response System (GISRS) regarding a threshold (fold change) for reporting, therefore an arbitrary threshold of ˃ three-fold half maximal effective concentration of a drug (EC_50_) was used to list PA AAS conferring reduced susceptibility to baloxavir (Table 1).

**Table 1:** Treatment-emergent and other amino acid substitutions in the polymerase acidic protein associated with reduced baloxavir susceptibility in vitro^a^

PA amino acid substitution	EC_50_ fold change^b^			
	A(H3N2)	A(H1N1)pdm09^c^	B	References
E23G	NA	4–5	ND	[3], [4], current study
E23K	6	5	< 3	[3], [4], [5], [7]
E23R	ND	NA	ND	[3]
A36V^d^	6	4	< 3	[5]
A37T	8	ND	ND	[3], [4], [5]
I38F	20	11	< 3	[3], [4], [5], [7]
I38M	14	13	8	[3], [4], [5], [14]
	10	ND	ND	current study
I38T	49–57	27	6	[3], [8], [4], [5] [7], [10]
I38L	­ND	8	ND	current study
E119D^e^	5	6	< 3	[5]
E199G	5	ND	ND	[3], [5]

NA: data not available; ND: not detected or tested; PA: polymerase acidic.

^a^ The Expert Working Group on Antiviral Susceptibility for the World Health Organization Global Influenza Surveillance and Response System has not yet issued guidance regarding a threshold (fold change) for reporting phenotypic test results for baloxavir. Arbitrary threshold (˃ three-fold EC_50_) was used to list PA amino acid substitutions that confer reduced susceptibility to baloxavir.

^b^ Fold change calculated by comparing EC_50_ of test virus to the reference value (e.g. control virus).

^c^ Reverse genetically engineered Influenza A(H1N1) viruses, A/WSN/33 and A/Puerto Rico/8/34 were also tested.

^d^ F36V in PA of influenza B virus.

^e^ E120D in PA of influenza B virus.

## Influenza surveillance based on codon-complete next generation sequencing

In accordance with Centers for Disease Control and Prevention (CDC) guidance, public health laboratories (PHLs) submit up to two viruses of each subtype (type A) and lineage (type B) to national surveillance, twice a month. Since the implementation of the Sequence First Initiative, all submitted viruses have been subjected to codon-complete next generation sequencing (NGS) of viral genomes [9] (Table 2). In addition, NGS data were obtained for 235 viruses submitted as part of enhanced antiviral surveillance in the 2017/18 season. All sequences were made public through the National Center for Biotechnology Information (NCBI) influenza virus resource and the Global Initiative on Sharing All Influenza Data (GISAID). To ensure accuracy, the sequences were curated to remove duplicate sequences for individual viruses (Table 2). A large subset of clinical specimens was used for virus isolation to conduct antigenic, antiviral and other analyses.

**Table 2:** Influenza viruses submitted to the United States national surveillance and subjected to next generation sequencing analysis, 2016/17 and 2017/18 influenza seasons

Virus	Influenza Season		Total
	2016/17	2017/18^a^	
A(H3N2)	1,594	1,570	3,164
A(H1N1)pdm09	383	967	1,350
B/Victoria	459	304	763
B/Yamagata	660	954	1,614
Total^b^	**3,096**	**3,795**	**6,891**
Specimen only	2,168	3,036	5,204
Isolate only	81	62	143
Both specimen and isolate	847	697	1,544

^a^ Included in the analysis were 235 viruses, 185 A(H3N2) and 50 A(H1N1)pdm09, collected from 12 US states for enhanced antiviral surveillance.

^b^ The total number of influenza viruses with polymerase acidic sequences downloaded from Global Initiative on Sharing All Influenza Data database; duplicate sequences were not counted.

Season 2016/17 dates are 1 October 2016–30 September 2017; season 2017/18 dates are 1 October 2017–30 September 2018.

## Polymerase acidic protein amino acid substitutions associated with reduced baloxavir susceptibility

Sequences were analysed for changes in the PA protein previously associated with reduced baloxavir susceptibility (Table 1). Of 6,891 viruses collected during 2016/17 and 2017/18 seasons (1 October 2016 to 30 September 2018), none contained the principal marker of baloxavir resistance I38T. Of 3,164 A(H3N2) viruses, A/Louisiana/49/2017 contained I38M. Among 1,350 A(H1N1)pdm09 viruses, A/Florida/20/2018 and A/Arizona/35/2018 had E23G, an AAS previously reported in A(H3N2) viruses [4]. Two other viruses, A/Illinois/37/2018 and A/Illinois/38/2018, had I38L; the effect of this AAS on baloxavir susceptibility has not been previously evaluated (Table 3). None of the type B viruses (n = 2,377) contained AAS of concern. All PA substitutions described above were present in both, clinical specimens and respective virus isolates.

**Table 3:** Viruses containing polymerase acidic amino acid substitutions that showed reduced baloxavir susceptibility, United States, 2016/17 and 2017/18 influenza seasons

Type/subtype	Virus name	Codon^a^	PA AA	HINT			FRA	
				EC_50_, nM Mean ± SD^b^	Fold change to control^c^	Fold change to median^d^	EC_50_, nM Average^e^	Fold change to control
A(H3N2)	A/Louisiana/50/2017	ATA	I38	1.33 ± 0.22	1	2	1.12	1
	A/Louisiana/49/2017	ATG	I38M	13.88 ± 2.25	10	17	11.75	11
A(H1N1)pdm09	A/Kentucky/13/2018	GAA	E23	2.39 ± 1.58	1	2	NT	NT
	A/Florida/20/2018	GGA	E23G	8.86 ± 1.62	4	6	NT	NT
	A/Texas/121/2018	GAA	E23	1.90 ± 0.43	1	1	NT	NT
	A/Arizona/35/2018	GGA	E23G	10.24 ± 2.78	5	7	NT	NT
	A/Illinois/08/2018	ATA	I38	1.61 ± 0.22	1	1	2.12	1
	A/Illinois/37/2018	CTA	I38L	13.50 ± 3.04	8	9	14.96	7
	A/Illinois/38/2018	CTA	I38L	12.95 ± 1.87	8	8	NT	NT

AA: amino acid; FRA: focus reduction assay; HINT: high-content imaging neutralization test; NT: not tested; PA: polymerase acidic; SD: standard deviation.

^a^ Underlined base indicates the nt change.

^b^ Mean and SD of ≥ three independent tests.

^c^ Fold change to EC_50_ of test virus compared with sequence-matched control virus. A/Texas/121/2018 and A/Arizona/35/2018 also contained E688G and E126D in PA, respectively.

^d^ Fold change to EC_50_ of test virus compared with baseline median values: 0.80 for A(H3N2) and 1.57 for A(H1N1)pdm09 (Table 4).

^e^ Average of two results.

An arbitrary threshold (˃ three-fold EC_50_) was used to report PA amino acid substitutions that confer reduced susceptibility to baloxavir.

Other PA substitutions were detected either as pure populations (I38V) or heterogeneous subpopulations (A37T/A, I38K/I, or E119K/E) (Supplemental Table S1). Notably, the heterogeneous subpopulation E119K/E was found in a virus isolate, but could not be detected in the respective clinical specimen, suggesting emergence of AAS during virus propagation.

## Baloxavir susceptibility testing by cell-based phenotypic assays

As molecular mechanisms of reduced susceptibility to baloxavir are not yet fully elucidated, it is necessary to supplement NGS analysis with phenotypic testing. A phenotypic reduction in susceptibility is expressed as a fold change compared with a reference EC_50_. Following approval of baloxavir in Japan, the baseline susceptibilities for seasonal viruses circulating in Japan were established using a focus reduction assay (FRA) [10].

Previously, Stevaert et al. suggested using a one-cycle infection assay to test PA inhibitors [11]. Recently, we developed a one-cycle infection assay, known as high-content imaging neutralization test (HINT), for antigenic analysis [12]. A single infection cycle is achieved by omitting trypsin, thereby preventing virus spread to neighbouring cells (Supplemental Figure S1). Virus-infected cells are detected by immunofluorescence. HINT was used in this study to establish type-/subtype-specific baseline EC_50_ values.

To this end, 116 viruses representing different subtypes (type A) and lineages (type B) circulating in the US during 2016/17 and 2017/18 seasons were tested (Table 4). EC_50_ values were similar to those reported for viruses tested using a plaque reduction assay [5]. As expected, type B viruses displayed ca four-fold greater EC_50_ compared with type A viruses. The consistency between HINT and plaque reduction assay was encouraging as EC_50_ values determined using a different assay, ViroSpot, appeared to be elevated [5].

**Table 4:** Baseline baloxavir susceptibility of seasonal viruses, United States, 2016/17 and 2017/18 influenza seasons

Virus	Number of viruses^a^	Baseline EC_50_ (nM)^b^		
		Range	Mean ± SD	Median
A(H3N2)	28	0.33–1.53	0.83 ± 0.30	0.80
A(H1N1)pdm09	34	0.86–2.86	1.58 ± 0.49	1.57
B Victoria	31	1.79–8.03	4.79 ± 1.48	4.82
B Yamagata	23	1.72–8.29	5.00 ± 1.50	4.92

SD: standard deviation.

^a^ Seasonal United States influenza viruses collected during May 2017–August 2018 were propagated in cell cultures and used for baseline median EC_50_ determination.

^b^ Viruses tested as single titration in a single assay to determine EC_50_.

Next, we tested 11 nasal washes from patients positive for influenza B (C_t_ values ranged 19.06–22.23). The EC_50_ values ranged from 2.5–6.7 nM, which was within the established baseline for type B virus isolates (data not shown).

Viruses flagged by NGS analysis were tested alongside their PA-matching counterpart (i.e. control viruses) (Table 3 and Supplemental Table S1). A/Louisiana/49/2017, containing I38M, displayed a 10-fold change in baloxavir susceptibility compared with the control virus A/Louisiana/50/2017, which is in agreement with a previous report for another A(H3N2) virus using a plaque reduction assay [5] (Table 1). We also tested this virus pair by a modified FRA [13]; an 11-fold change in baloxavir susceptibility was detected. Furthermore, Omoto et al. independently tested these viruses using a plaque reduction assay and reported a 13-fold change [14]. Therefore, the similar results from HINT and multi-cycle infection assays re-enforce the utility of HINT for testing baloxavir susceptibility.

The new substitution, I38L, conferred an eight- and seven-fold change compared with the control virus in the HINT and FRA assays, respectively. Two A(H1N1)pdm09 viruses carrying E23G displayed a four–five-fold change by HINT (Table 3). When fold changes were calculated using type- and subtype-specific median EC_50_ values (Table 4), the results were generally in agreement. However, a fold change for A/Louisiana/49/2017 I38M virus was higher when calculated against the median EC_50_ , because the EC_50_ of the matching control virus was ca two-fold above the median (Table 3 and 4). The presence of heterogeneous subpopulations had no apparent effect on baloxavir susceptibility (Supplemental Table S1); nevertheless, their relevance cannot be ruled out at this time. As expected, I38V conferred one–two-fold change compared to control viruses, which was previously reported [5].

Combining NGS and HINT data from two seasons, the frequency of viruses displaying reduced baloxavir susceptibility (˃ three-fold change) was low: 0%, 0.032% and 0.3%, for type B, A(H3N2) and A(H1N1)pdm09 viruses, respectively. Similar to a previous report, A(H1N1)pdm09 viruses exhibited a higher frequency of PA amino acid polymorphism [11].

Out of 26 A(H1N1)pdm09 viruses submitted from Illinois, two contained I38L, A/Illinois/37/2018 and A/Illinois/38/2018, which were identical on a nt level. Both were collected on 8 February 2018 from school age males residing in the same county. Because natural polymorphism at this residue is rare, we investigated for potential transmission.

The CDC requested additional A(H1N1)pdm09 viruses collected in this state during the 2017/18 season. Forty-one additional A(H1N1)pdm09 positive specimens were available for evaluation. To expedite testing, the pyrosequencing assay [15] was used to test for AAS at residue 38 (Supplemental Figure S2). Results were obtained for 30 specimens, all of which lacked I38L. The combined results from NGS and pyrosequencing estimated the rate of detection of I38L to be 3.6% (2/56) for Illinois and 4.3% (2/47) for this particular county.

## Discussion and conclusion

In this study, we established the methodology for monitoring baloxavir susceptibility in the US ahead of the drug entering the national market. As NGS analysis has become the cornerstone of influenza surveillance, it created a platform for monitoring resistance for all FDA-approved anti-influenza therapeutics. The frequency of AAS associated with reduced baloxavir susceptibility should be determined by type and subtype. This will allow for accurate inter-seasonal comparisons, as different types and subtypes predominate season to season. In the event of suspected emergence of resistance, either NGS or pyrosequencing could support epidemiological investigations in a timely manner. Pyrosequencing has been employed by many US PHLs to conduct enhanced antiviral surveillance, so this assay can readily be implemented for detecting baloxavir resistant viruses. Phenotypic assays detect viruses with reduced drug susceptibility regardless of underlying mutations. Here, HINT was successfully applied to establish the baseline susceptibility of seasonal viruses. Moreover, we have shown that clinical specimens could be tested directly by HINT, which could expedite surveillance.

Overall, in the 2016/17 and 2017/18 seasons the frequency of AAS associated with reduced susceptibility to baloxavir was low. For the current 2018/19 season, we analysed PA sequences of 384 US viruses (accessed from GISAID on 11 January 2019) and found no AAS at positions of concern. Additionally, we tested 73 representative viruses by HINT and all EC_50_ values were within their respective type/subtype baseline (data not shown).

The antiviral testing algorithm described above will be applied to both foreign and domestic viruses. In collaboration with the Association of Public Health Laboratories, CDC aims to advance antiviral surveillance by implementing HINT at designated National Influenza Reference Centers. To assist in baloxavir susceptibility testing at the WHO National Influenza Centers and other laboratories, a CDC panel of reference viruses with reduced susceptibility to baloxavir will be made available from the International Reagent Resource [16]. As baloxavir is expected to be more commonly prescribed in Japan and the US in the coming seasons, close monitoring of resistance is necessary to inform public health policies regarding antiviral use.

## Supplementary Data

643000 Supplementary material
