# Supplementary material for: Assessing baloxavir susceptibility of influenza viruses circulating in the United States during the 2016/17 and 2017/18 seasons
Source: Euro Surveill. 2019 Jan 17;24(3):1800666. doi: 10.2807/1560-7917.ES.2019.24.3.1800666 (PMC6344838; doi:10.2807/1560-7917.ES.2019.24.3.1800666)
Supplement: Supplementary material [file 1800666_GUBAREVA_Supplementary.pdf]

SUPPLEMENTARY MATERIAL for “Assessing baloxavir susceptibility of influenza viruses circulating in the United States during the 2016/17 and 2017/18 seasons”

Larisa V Gubareva[1], Vasiliy P Mishin[1], Mira C Patel[1,2], Anton Chesnokov[1], Ha T Nguyen[1,2], Juan De La Cruz[1,2], Sarah Spencer[1], Angela P Campbell[1], Mallory Sinner[3], Heather Reid[3], Rebecca Garten[1], Jackie M Katz[1], Alicia M Fry[1], John Barnes[1], David E Wentworth[1]

1. Influenza Division, National Center for Immunization and Respiratory Disease, Centers for Disease Control and Prevention (CDC), Atlanta, United States of America

2. Battelle, Atlanta, United States of America

3. Illinois Department of Public Health, Springfield, United States of America

Correspondence: Larisa V Gubareva (lgubareva@cdc.gov)

Supplementary document containing information on (1) polymerase acidic amino acid substitutions of concern with no evident effect on baloxavir susceptibility, (2) the workflow of the high-content imaging neutralization test (HINT) and (3) pyrosequencing results showing nucleotide changes associated with reduced susceptibility to baloxavir.

This supplementary material is hosted by Eurosurveillance as supporting information alongside the article “Assessing baloxavir susceptibility of influenza viruses circulating in the United States during the 2016/17 and 2017/18 seasons” on behalf of the authors who remain responsible for the accuracy and appropriateness of the content. The same standards for ethics, copyright, attributions and permissions as for the article apply. Eurosurveillance is not responsible for the maintenance of any links or email addresses provided therein

Supplemental Table S1. Viruses containing polymerase acidic amino acid substitutions that did not affect baloxavir susceptibility, tested by HINT, United States, 2016/17 and 2017/18 influenza seasons

Type/subtype	Virus name	Codon[a]	PA AA (% substitution)[a]	EC_{50}, nM Mean ± SD[c]	Fold change to control[d]	Fold change to median[e]
A(H3N2)	A/Maine/17/2017	GCA	A37	0.79 ± 0.13	1	1
	A/Maine/15/2017	G/ACA	A37T/A (44)	0.63 ± 0.10	1	1
	A/Hawaii/26/2017	ATA	I38	0.36 ± 0.12	1	1
	A/Hawaii/28/2017	AT/AA	I38K/I (27)	0.54 ± 0.12	2	1
	A/West Virginia/06/2018	GAA	E119	0.30 ± 0.09	1	1[f]
	A/California/148/2017	G/AAA	E119K/E (40)	0.50 ± 0.16	2	1
	A/Hawaii/67/2016	ATA	I38	1.17 ± 0.20	1	1
	A/Hawaii/89/2016	GTA	I38V (100)	0.51 ± 0.13	1[g]	1
A(H1N1)pdm09	A/Connecticut/28/2016	ATA	I38	1.90 ± 0.52	1	1
	A/California/153/2016	GTA	I38V (100)	4.09 ± 1.73	2	3
B Yamagata	B/Texas/98/2017	ATC	I38	9.17 ± 4.30	1	2
	B/Oregon/07/2018	GTC	I38V (100)	9.40 ± 1.28	1	2

AA: amino acid; PA: polymerase acidic; SD: standard deviation.

[a] Underlined base indicates the nt change.

[b] Number in parentheses indicate % containing substitution determined by next generation sequencing (20-25% as threshold)

[c] Mean and SD of ≥ three independent tests.

[d] Fold change to EC_{50} of test virus compared to respective sequence-matched control virus. A/California/153/2016 and B/Oregon/07/2018 also contained H144Y and K605R in PA, respectively.

[e] Fold change to EC_{50} of test virus compared with baseline median values: 0.80 for A(H3N2), 1.57 for A(H1N1)pdm09 and 4.92 for B Yamagata (Table 4).

[f] Fold change < 0.5 is shown as 1.

[g] Fold change < 0.5 is shown as 1.

PA amino acid substitutions causing ≤ three-fold change in EC_{50} are not considered as affecting baloxavir susceptibility.

Supplemental Figure S1. High-content imaging neutralization test (HINT) workflow

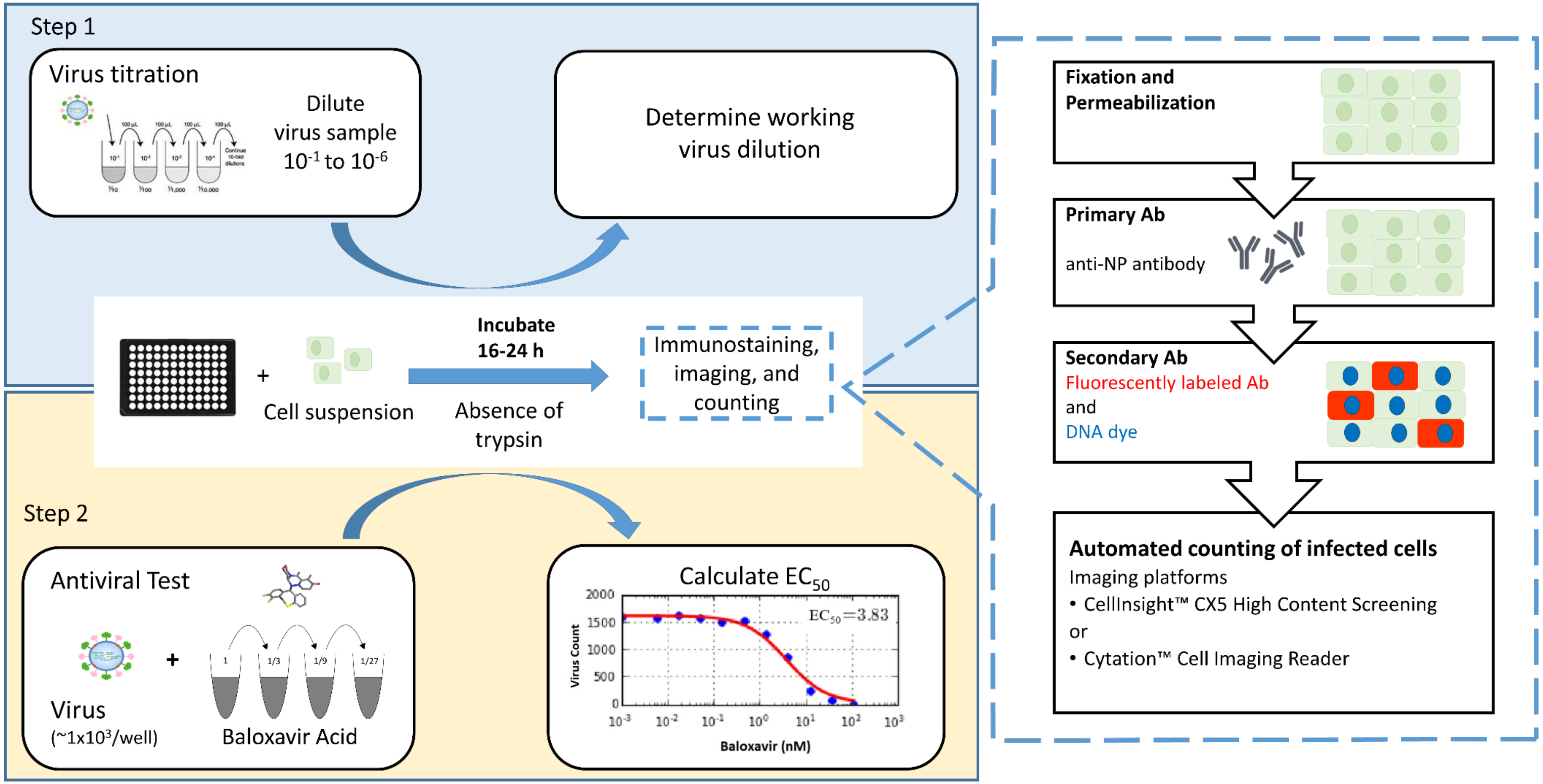

Supplemental Figure S2. Pyrosequencing readouts of polymerase acidic amino acid residue 38 for influenza A(H1N1)pdm09 and A(H3N2) viruses

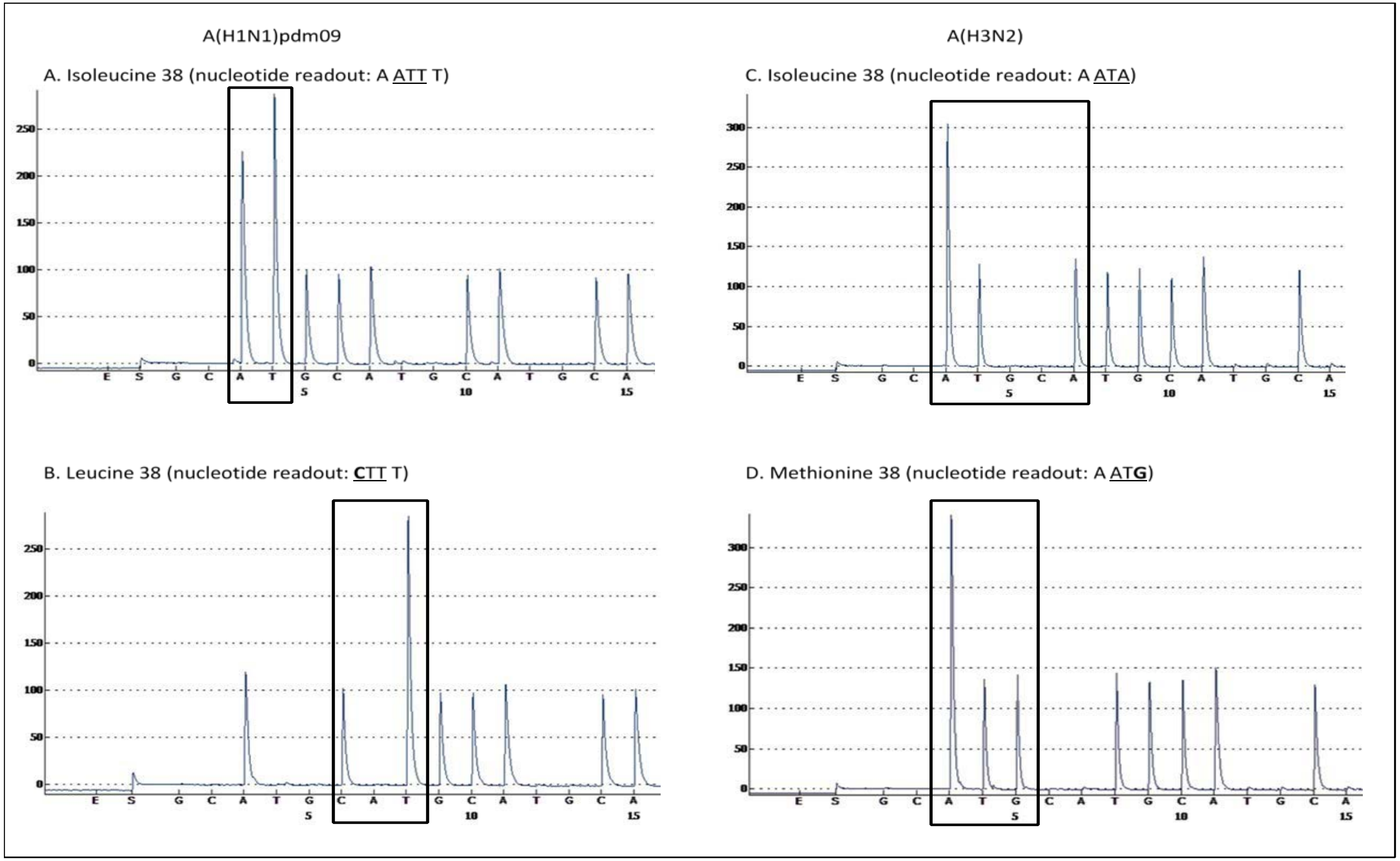

Pyrosequencing assay conducted as previously described [reference 15].

Forward RT-PCR primer nt58, 5’- GCA ATG AAA GAR TAT GGG G-3’.

Reverse RT-PCR primer nt280, Biotin-5’-TAC TGT TYA CYA CTG TCC AGG CCA-3’.

Sequencing primers: A(H1N1)pdm09 nt91, 5’-GAA ACT AAT AAG TTT GCT GC-3’; A(H3N2) nt95, 5’-CC AAC AAA TTT GCA GC-3’.

Black box designates nt readout containing the PA-38 codon. Sequence readouts: A) A ATT TGC ACA CA; B) A **C**TT TGC ACA; C) A ATA TGC AC; D) A AT**G** TGC AC. Underline designates nt triplet encoding amino acid at residue 38 of PA protein. Bold indicates nt change.
